# Dependent, but not Perfectionistic, Dysfunctional Attitudes Predict Worsened Mood and Appraisals after Emotional Support from a Romantic Partner

**DOI:** 10.3389/fpsyg.2016.01502

**Published:** 2016-10-13

**Authors:** Steven A. M. Felix, Christine I. Hooker

**Affiliations:** ^1^Department of Psychology, Harvard UniversityCambridge, MA, USA; ^2^Department of Psychiatry, Rush UniversityChicago, IL, USA

**Keywords:** emotional support, dysfunctional attitudes, dependency, depression, enacted support

## Abstract

**Background:** Receiving emotional support from a romantic partner often leads to emotional costs via negative appraisals about the self and one's relationship, but it is unclear whether certain individuals are more susceptible to these costs. We evaluate whether the presence of perfectionistic and dependent dysfunctional attitudes leads to more negative effects of receiving emotional support from a romantic partner.

**Methods:** Twenty-nine couples (27 men, 31 women; mean age 24.5) completed the Dysfunctional Attitudes Scale and then a daily online questionnaire by recording their mood, appraisals, and received emotional support. Mixed-effects regressions were used to evaluate whether perfectionistic and dependent dysfunctional attitudes moderated the relationship between emotional support receipt and subsequent mood and appraisals.

**Results:** Perfectionism did not interact with emotional support but exerted a main effect of increasing negative moods and appraisals. Dependency interacted with emotional support such that those with more dependent attitudes reported greater negative next-day moods and appraisals as a function of emotional support.

**Conclusions:** Individuals with dependent, but not perfectionistic, dysfunctional attitudes are more likely to experience emotional and cognitive costs after receiving emotional support. These costs may stem from activation or exacerbation of the attitudes specific to dependency, including need for acceptance, support, and approval of others.

## Background

The cognitive diathesis-stress model of depression posits that chronic negative, dysfunctional thinking acts as a vulnerability to depression in the context of stressful life events. Life stressors repeatedly activate and exacerbate dysfunctional thoughts, leading to negative beliefs about the self, the world, and the future, which ultimately culminate in depression (Beck, 1967; Weissman and Beck, 1978). Effective interventions for preventing or treating depression, therefore, are those that help cognitively vulnerable individuals reduce their negative thinking when they encounter life stress.

Although social support has been proposed as one such intervention that can buffer the ill effects of stress by altering negative appraisals of events (Cohen and Wills, 1985), evidence for the buffering hypothesis is weak and growing research indicates that support can often worsen negative appraisals (Rafaeli and Gleason, 2009). In this paper, we examine whether susceptibility to these negative moods and appraisals after receiving emotional support varies as a function of a person's dependent and perfectionistic dysfunctional attitudes.

### Dysfunctional attitudes

According to Becks' cognitive theory of depression (Beck, 1967), certain individuals have negative, depressogenic schemata that are activated or exacerbated by stressful life events. These depressogenic schemata are organized into dysfunctional attitudes: rigid, if-then contingencies for evaluating happiness and self-worth (Weissman and Beck, 1978; Olinger et al., 1987). These contingencies have often been grouped into two categories: perfectionistic and self-critical attitudes (e.g., “If I fail partly, it is as bad as being a complete failure”) and dependent attitudes (e.g., “I am nothing if a person I love doesn't love me”; de Graaf et al., 2009). Activation of these dysfunctional attitudes by stressful events or negative mood leads to negatively-biased information processing characterized by distorted thinking (e.g., biased interpretations, overgeneralizations, all-or-nothing thinking), negative self-evaluations, and rumination. Through these mechanisms, depressogenic schemata and life stress foster the negative cognitive triad—negative views of the self, the world, and the future—which Beck hypothesized was the final pathway to depression. It is important to note that Beck also theorized that this pathway was specific: Dysfunctional attitudes predispose individuals to depressive, but not anxious, symptoms.

Tests of dysfunctional attitudes as a cognitive diathesis to depression have supported Beck's theory, generally finding that individuals with greater dysfunctional attitudes adjust poorly to both major and minor stressors: Cross-sectional studies demonstrate that dysfunctional attitudes moderate the relationship between number of life stressors and levels of dysphoria and depression such that stressors are more strongly linked to depression among individuals with greater dysfunctional attitudes (Wise and Barnes, 1986; Olinger et al., 1987; Kuiper et al., 1988). Longitudinal studies have provided a more valid test of Beck's theory, finding that dysfunctional attitudes prospectively predict increases in depression symptoms after negative events in children, adolescents, and adults (Zuroff et al., 1990; Joiner et al., 1999; Abela and D'Alessandro, 2002; Abela and Sullivan, 2003; Abela and Skitch, 2007). Some of these studies have also tested and found support for the mediation component of Beck's theory: That the increases in depression among individuals with high dysfunctional attitudes after stressful life events is mediated by increases in automatic negative thoughts about the self, the world, and the future (Joiner et al., 1999; Abela and D'Alessandro, 2002). The findings of Joiner et al. (1999) also support the specificity hypothesis, as the interaction of dysfunctional attitudes with stressful life events lead to increases in depressive but not anxious cognitions. Together, this research supports the idea that dysfunctional attitudes make individuals more vulnerable to the negative effects of life stress, ostensibly via activation of automatic depressive cognitions about the self, others, and the future.

### Support as a stress buffer

Based on Beck's cognitive model of depression, many researchers have looked for protective factors that might counteract dysfunctional thinking in stressful situations. Social support is one such factor that has garnered much attention for its theoretical potential to buffer against depression-inducing stress, possibly through reduction or modification of negative appraisals. Research investigating this *buffering hypothesis* (Cohen and Wills, 1985) has generally focused on two distinct and non-interchangeable social support constructs: perceived support and received (or enacted) support (Barrera, 1986).

The vast majority of research on social support and depression has investigated perceived support, which is defined as the extent to which a person believes that he or she is connected to resources and people who would be willing to provide adequate emotional and instrumental support should the need arise (Barrera, 1986). Thus, it is a global, subjective appraisal that one is loved, cared for, and supported and is likely influenced by a variety of different processes and constructs, including one's current psychological state (e.g., distress level, depression), personality (e.g., neuroticism, trait loneliness), and other idiosyncratic evaluative processes (Cobb, 1976; Paykel, 1994; Haber et al., 2007). Investigations of perceived support have consistently demonstrated better outcomes for individuals who perceive greater support, including lower likelihood of onset and recurrence of depressive episodes, as well as reduced severity and improved recovery from it (for review, see Ibarra-Rovillard and Kuiper, 2011). Unfortunately, the mechanisms for these effects are unclear, in part because perceived support taps so many different constructs and processes. What is clear, however, is that these effects are not likely transmitted through individual support transactions: Not only are perceived and received support are weakly related (Haber et al., 2007), they have been associated with distinct outcomes.

Received support is defined as specific supportive behaviors provided to a recipient. Received support has been operationalized numerous ways. In the depression literature, which has mostly been concerned with understanding whether high levels of support buffer people against stress, received support is typically measured using monthly checklists to quantify the amount of support received. These checklists, such as the Inventory of Socially Supportive Behaviors (Barrera et al., 1981), ask respondents to mark the frequency with which they received various specific support behaviors (e.g., “Listened to you talk about your private feelings”, “Was right there with you (physically) in a stressful situation”).

The social support literature, in contrast, has been more focused on understanding the specific psychological effects of *individual* support transactions. Consequently, this research has tended to operationalize received support in ways that capture discrete instances of support and assesses psychological outcomes before and after support receipt, for instance, via laboratory observation (e.g., Howland and Simpson, 2010), laboratory experimentation, (e.g., Bolger and Amarel, 2007), or daily diary measurement (e.g., Bolger et al., 2000; Shrout et al., 2006; Gleason et al., 2008). In stark contrast to studies of perceived support and checklist studies measuring total quantities of received support, studies of received support using these methods have yielded clear and consistent evidence that received support, particularly received emotional support, paradoxically leaves support recipients feeling worse than when they do not receive support.

For instance, using a 4-week daily diary among romantic couples in which one member of each couple was about to take the Bar Exam, Bolger et al. (2000) measured daily anxiety and depression symptoms along with receipt of emotional support. Emotional support was assessed by a dichotomous question (yes/no) asking participants whether their partners had “listened to or comforted” them each day. They found that self-reports of receiving emotional support predicted greater *next day* anxiety and depression. Using the same diary method with the addition of measuring practical support (i.e., concrete behaviors aimed at trying to help solve a problem), Shrout et al. (2006) found that self-reports of receiving emotional support, but not practical support, were associated with increased anger, anxiety, and depression.

The authors argue that receiving emotional support can have unexpected emotional and self-esteem costs. Emotional support may, for instance, leave a person feeling inefficacious or incompetent to cope with his or her problems independently. Emotional support may also lead to negative psychological effects by fostering feelings of relationship imbalance or of indebtedness to one's partner. In a diary study using the same methods as Bolger et al. (2000), Gleason and colleagues (2008) found that self-reports of receiving support were associated with greater distress, but only when the respondent did not also provide support to his or her partner. When the respondent reported both giving and receiving support, emotional support receipt was associated with reduced personal distress.

Forms of emotional support, such as empathy, validation, and non-directive listening, may also contribute to these costs by confirming negative views instead of challenging them or by encouraging excessive negative attention and elaboration on the stressors or negative appraisals. When observing couples providing support in the laboratory, Howland and Simpson (2010) found that emotional support was harmful when it “focuses on the partner and his/her problem and draws attention to the partner/problem” and “focuses on the partner's limitations and how upsetting/stressful the issue is”, and beneficial when it did the opposite (p. 1881).

There is also evidence of individual differences in the costs of emotional support. Analyzing the random effects in their mixed effects models, Gleason et al. (2008) detected significant heterogeneity in people's response to emotional support, with some individuals reaping only benefits (greater partner closeness and reduced distress) and others only harms (reduced closeness and greater distress). The authors tested self-esteem and relationship satisfaction as potential moderators but found no evidence for relationship satisfaction and only some evidence for self-esteem. Shrout et al. (2010) similarly found significant individual variation in people's response to emotional support, emphasizing the importance for future research to identify individual differences that better explain the observed heterogeneity.

These studies have important implications for understanding the relationship between emotional support and dysfunctional attitudes. First, received emotional support may not in fact provide stress-buffering effects, at least not through reduction of dysfunctional thinking. Emotional support often increases or exacerbates negative appraisals about the self and others (e.g., “I can't cope alone”, “I'm a failure”, “I'm indebted to my partner”), many of which map directly onto the dysfunctional attitudes that make individuals vulnerable to depression (e.g., “If a person asks for help, it is a sign of weakness”, “If I fail partly, it is as bad as being a complete failure”, “If you don't have other people to lean on, you are going to be sad”). The parallels in these appraisals suggest that support may be particularly problematic for individuals with more dysfunctional attitudes; support may paradoxically activate in such people the exact dysfunctional thoughts and appraisals that predispose them to dysphoria and depression. In other words, individuals with greater dysfunctional attitudes may be more vulnerable to the cognitive and emotional costs associated with receiving support. Given that the negative effects of emotional support seem to be driven by cognitive processes, dysfunctional thinking may be a stronger indicator than self-esteem of a person's vulnerability to the negative thoughts arising from support transactions.

### Current study

In the present study, we evaluate this hypothesis by assessing how dysfunctional attitudes interact with received emotional support in daily life to affect three outcomes commonly associated with depression and depressive symptomatology: depressed mood, appraisals of general well-being, and appraisals of perceived support (i.e., the more general feeling that one is loved, supported, and cared for). Couples from the community completed a dysfunctional attitudes questionnaire and then completed a 3-week daily diary assessing moods, attitudes, and received emotional support in their significant interactions. Because dysfunctional attitudes are normally distributed in community samples (de Graaf et al., 2009) and theoretically contribute continuous risk for dysphoria and depression, a community sample provides an adequate population in which to test our hypothesis.

There are a few important differences in our methodology compared to other diary studies. Prior diary studies assessed the occurrence (yes/no) of emotional support on each day of participation. Though this approach may validly identify days in which support was received, it fails to provide important information about days in which support was not received. Specifically, this approach cannot distinguish between an uneventful day in which no support was needed or exchanged, and a day that included a negative and unsupportive interaction with one's partner. The difference between these two days would be expected to influence mood and well-being outcomes in significantly different ways, but they are confounded in this approach. This method also fails to assess the quality and context of the support that was exchanged. Support can be of varying qualities and can often be accompanied by criticism or conflict (Rafaeli and Gleason, 2009), and prior studies cannot rule out whether other aspects of support exchange, such as potential criticism, contribute to the observed paradoxical results. To address these issues, in the current study we assess support only when participants report having significant or meaningful interactions with their partners. Furthermore, when participants do report such interactions, we assess the *extent* of emotional supportiveness as well as other characteristics such as criticism and conflict. This approach allows us not only to capture greater variability in participants' perceptions of emotional support in an interaction, but also to characterize the context in which support was received or not received. In turn, this may allow for generalizability of findings to a wider variety of couples' interactions.

Based on the findings from Gleason et al. (2008), we predicted that trait dysfunctional attitudes (consisting of dependency and perfectionism subscales) would moderate the relationship between ratings of received emotional support during couples' interactions and next-day well-being such that greater received emotional support would predict reduced well-being for those with greater dysfunctional attitudes and increased well-being for those with fewer dysfunctional attitudes. We also predicted that dysfunctional attitudes would exert a main effect of reducing well-being.

## Methods

This study was approved by and conducted according to the standards of the Harvard University Committee on the Use of Human Subjects.

### Participants

Thirty-eight couples (72 individuals) were initially recruited to participate in a diary study via online advertisements on Craigslist. Interested participants were eligible so long as they were over 18 and had been in their relationship for more than 3 months, and did not have any current psychological disorder, since current symptomology and treatment of mental illness, particularly depression, could confound interpretation of results. Currently-healthy participants with a history of mental illness were not excluded as these individuals are likely to exhibit the vulnerability of interest without posing as much of a risk of confounding. Current or history of mental illness was determined using the Mini International Neuropsychiatric Interview (M.I.N.I.), English Version 5.0.0 (Sheehan et al., 1998).

Of the 38 couples that were initially recruited, 4 were immediately excluded due to current psychological disorder, 4 terminated their participation early (either before or during the diary), and 1 was terminated early by the researchers due to poor study compliance. The final sample includes data from 29 couples (27 males, 31 females), for a total of 58 individuals. The mean age was 24.5 years. Thirty 6% of the sample had a high school diploma or some college, and 64% held a bachelor's degree or higher. Seventy five percent were white; 10%, Asian; 3.5%, Hispanic; 2%, African American; 5%, Mixed; and 3.5%, other. Nine participants had a history of mental illness, with 8 reporting history of depression, and 4 reporting history of anxiety (3 of these reported both). Couples had been together for an average of 2.13 years (median = 1.87 years). Sixty two percent of couples were dating, 28% living together, 3% engaged, and 7% married. Data on socioeconomic status was not collected.

### Diary procedure

All couples completed a measure of dysfunctional attitudes before beginning the diary. Participants were instructed to complete the online diary questionnaire every night as close to bedtime as possible for 21 consecutive days. The online diary was not adapted for smartphone use, so all responses were made by computer. Participants were to complete the diary alone, without input from their partners and were instructed not to talk about their responses to the diary with their partners while they were still participating in the study.

### Daily ratings of mood, appraisals, and partner interactions

Each day participants rated the following items/statements concerning their moods and beliefs. All responses were measured on 5-point Likert scales ranging from 1: “Not at all” to 5: “Extremely (often)”.

#### Depressed mood

Participants' depressed mood was measured using a modified version of the depression items of the Profile of Mood States (McNair and Lorr, 1964). In addition to having participants rate the extent to which they felt “sad”, “hopeless”, and “discouraged”, we also asked participants to rate whether they felt “irritable”, “lonely”, and “isolated”, as these are also common feelings related to depression. Responses for these 6 items were averaged together to form a single index of depressed mood (Cronbach's α = 0.84).

#### Overall well-being

Well-being has been conceptualized as including feelings of competence, autonomy, self-esteem, and relatedness (Reis et al., 2000). As such, participants rated their agreement with 16 items tapping these constructs (e.g., “I felt as though I had the ability to solve my own problems”, “I felt as if I were free to do what I wanted or needed to do today”, “I felt good about myself today”, and “I felt connected to people I interacted with today”, respectively). Participants also rated 3 items tapping life satisfaction (e.g., “I felt content with my life today.”). Although it was our original intention to treat each of these constructs separately, initial analyses indicated that all of these constructs were highly correlated, so we decided to combine all 19 of these items into a single composite as a global representation of individuals' psychological well-being. Alpha reliability was excellent (α = 0.96).

#### Perceived support

Perceived support was operationalized as the extent to which participants generally felt “loved”, “valued”, “accepted”, and “supported” each day. Similar approaches have been used in diary studies to assess constructs like partner closeness (e.g., Gleason et al., 2008). Participants did not rate how much they felt supported by any particular person, but rather, how much they felt these emotions generally that day. Responses for these 4 items were averaged together (Cronbach's α = 0.90).

#### Partner interactions

Each day, we asked respondents whether or not they had engaged in “a meaningful or emotional interaction, discussion, disagreement, or conflict” with their partners that day, such as “talking about a worry, anxiety, or fear; sharing or processing a personal experience or event that happened recently; talking about something you were angry or annoyed about; talking about something you were sad or upset; a disagreement or argument over a topic that was personally meaningful”. The purpose of this constraint was to ascertain whether participants had interactions in which emotional support is typically desired and/or received. If participants responded “yes”, they were then presented with various questions assessing the emotional supportiveness of the interaction and other important characteristics.

##### Emotional support

Consistent with major theories of emotional support (e.g., Cutrona and Russell, 1990; Burleson, 2003), we operationalized received emotional support as perceptions that one's partner cared for him/her (“did she/he genuinely want to understand your feelings, experiences, or perspective, even if they were negative or unpleasant?”), empathized with and understood him/her (“was your partner able to see things through your eyes or from your point of view?”), validated him/her (“did your partner make you feel like your feelings were reasonable and made sense?”), and demonstrated a desire to help (“did your partner feel for you and want to help you?”). A total of nine-items assessing these aspects of emotional support were averaged together (Cronbach's α = 0.95).

##### Other characteristics

Participants also rated whether the interaction was conflictual (“To what extent would you describe this interaction as a conflict or disagreement?”) and whether or not they perceived criticism (“To what extent did your partner give you negative or critical feedback, advice, or direction?”). To assess the severity of the stressor that prompted the interaction, participants rated the extent to which they felt negative emotions about the topic (e.g., sad, angry, guilty, anxious). Of these, the highest rating was used as an indicator of severity of the instigating stressor.

### Individual differences and covariates

#### Dysfunctional attitudes

The Dysfunctional Attitudes Scale Form A (DAS-A; Weissman and Beck, 1978) is a 40-item, 7-point Likert scale assessing two domains of dysfunctional attitudes: perfectionism/performance evaluation and dependency/need for intimacy and affiliation (de Graaf et al., 2009). Analyses were conducted on both the entire scale (α = 0.91) as well as on the perfectionism and dependency subscales (11-items, α = 0.83, and 5-items, α = 0.74, respectively) delineated by de Graaf et al. (2009). One item (#27) was excluded from the dependency subscale as it was uncorrelated with the other items (*r*s = −0.15–0.08, all *ns*) and significantly reduced internal reliability (with #27, α = 0.65). All analyses were completed both with and without item #27, and no significant differences in our results were observed. The analyses presented in this paper exclude item #27 from the composite.

#### Covariates

In order to control for differences in peoples exposure to stressors, we created an index of recent stress in the days preceding our daily outcome measures. Participants reported 3 potential sources of stress each day: significant interactions with a partner (described above), significant interactions with a non-partner, and any miscellaneous negative events a respondent had experienced that day that were not captured by these two social interactions. For each of the two interactions, the stressfulness of the interaction was operationalized as the extent to which it was described as a conflict (on a 1–5 scale). For the negative event (respondents had the option to report 1 negative event per day), respondents rated how stressful the event was on a 1–5 scale.

To create an index of *recent* stress, we first created a *daily* stress score by summing the conflict scores from the two possible interactions with the severity score of the possible negative event. This created a daily stress score with a minimum of 0 and a maximum of 15. To create the *recent* stress index, we summed the stress score for a given day with the stress scores for the preceding 2 days. For example, the recent stress score for Wednesday consists of the summed daily stress scores of Monday, Tuesday, and Wednesday. Thus, recent stress scores could range from 0 to 45.

Gender, relationship satisfaction, relationship length, and history of mental illness were all tested as covariates, but none changed the parameter estimates of interest or their significance. So, for simplicity, the analyses presented in this paper do not include these variables.

### Analyses

All statistical analyses were conducted in R (R Core Team, 2014) using the *lme4* package for linear mixed-effects models (Bates et al., 2014), which allow us to account for the fact that our data took a non-independent, hierarchical structure: Daily responses were nested within individuals, which were nested within couples. Mixed-effects models account for such nested data by estimating a random intercept, which accounts for the variance in daily outcomes explained by individuals (level 2) and couples (level 3). Significance testing of fixed effects was conducted using the *lmerTest* package in R (Kuznetsova et al., 2015), which conducts an *F*-test using the denominator degrees of freedom calculated by the Kenward-Roger approximation method (Kenward and Roger, 1997).

Lagged outcomes were used in all analyses, such that received emotional support on day *t* would predict outcomes on day *t* + 1. Consistent with prior dyadic diary studies, we evaluated relationships between received emotional support and only *next day* outcomes (or, subsequent time points) in order to avoid confounding caused by measuring independent and dependent variables at the same time point. Assessing current mood and perceptions of support received earlier that day may lead to a biased measure of received support, as current mood could affect how one remembers, interprets, or reports on the social interaction.

For a given outcome in each analysis (e.g., depressed mood on day *t* + 1), we controlled for that response variable on day the prior day (e.g., depressed mood on day *t*). This converts the dependent variable to a measure of residualized change in the outcome from day *t* to day *t* + 1 (Bolger et al., 2000). Furthermore, using our index of recent stress, we controlled for the combined stress on days *t* − 1, *t*, and *t* + 1 (i.e., stress experienced on the same day as the dependent variable, as well as the two proceeding days).

To test our main hypotheses, we included the main effects for received emotional support and dysfunctional attitudes, as well as the interaction term between the two. To remove the influence of trait-level (i.e., level 2) variability on received emotional support, the emotional support variable was centered around each individual's own mean emotional support rating (Peugh, 2010). Dysfunctional attitudes was grand mean centered for interpretability. To understand any significant interactions, simple slopes analyses were conducted by evaluating the effect of emotional support on the outcome at two levels of dysfunctional attitudes: one standard deviation above and below the sample mean (Aiken and West, 1991). The significance of these slopes was determined by bootstrapping 95% confidence intervals with 1000 replications using the *boot* package in R (Canty and Ripley, 2015).

## Results

### Descriptives

Diary compliance was excellent. Excluding invalid responses, participants completed an average of 19.78 diary entries (*SD* = 1.64, Median = 20), leading to a total of 1147 daily observations across the entire sample. Table 1 provides descriptive statistics for each of our primary variables. Sample means for the dysfunctional attitudes scale were similar to the means reported by individuals in the general population (de Graaf et al., 2009). Based on norms for the general population (including both depressed and non-depressed individuals), 10 participants scored in the high range for dysfunctional attitudes; 17 above average; 5 average; 14 below average; and 10 low, indicating symmetrical distribution of dysfunctional attitudes. The two subscales were moderately correlated (*r* = 0.53, *p* < 0.001), and perfectionism was more highly correlated with the full scale DAS-A (*r* = 0.90, *p* < 0.001) than was dependency (*r* = 0.76, *p* < 0.001).

**Table 1:** **Means, standard deviations, and ranges for dysfunctional attitudes, daily moods and appraisals, recent stress, and characteristics of partner interactions**.

	**Mean**	***SD***	**Scale Range**
**Dysfunctional attitudes**			
Full scale	115	27.38	40–280
Dependency	16.59^a^	5.66	5–35
Perfectionism	25.45	9.77	11–77
**Daily mood and appraisals**^b^			
Depressed mood	1.49	0.39	1–5
Well-being	3.63	0.66	1–5
Perceived support	3.89	0.62	1–5
**Recent stress**^b^	3.41	1.95	1–45
**Partner interactions**^b^			
Topic distress	3.14	1.18	1–5
Conflict	2.09	1.1	1–5
Criticism	1.9	0.94	1–5
Emotional support	3.43	0.99	1–5

a For comparison to norms (de Graaf et al., 2009), descriptives for the dependency subscale including item #27 (i.e., with all 6 items proposed by de Graaf et al., 2009) are M = 22.43, SD = 5.79, range = 6–42.

b Descriptives for diary items were calculated by first calculating the average response for each participant, and then taking the average of the resulting values.

Participants reported having significant interactions with their partners on 235 days, with an average of 4 interactions per person (*SD* = 3.09, Median = 3). Table 1 provides descriptive statistics of the levels of emotional support, conflict, and criticism in the interaction, as well as levels of distress related to the topic of the interaction. Concerning the topics of participants' interactions, participants engaged with their partners about moderately distressing topics. And on average, participants characterized these interactions as moderately supportive and only somewhat conflictual.

We also evaluated whether perfectionistic or dependent dysfunctional attitudes were related to levels of recent stress and to characteristics of the couples' interactions. To do this, we conducted mixed effects models in which we regressed recent stress^1^ and four interaction characteristics (e.g., distress related to the topic, criticism, conflict, and emotional support) on dependency and perfectionism. Table 2 displays the regression coefficients for each model. Dependency was unrelated to recent stress and all of the interaction characteristics. Perfectionism was related only to lower emotional support [*F*_(1, 46.88)_ = 4.81, *p* = 0.03], but not recent stress or any other interaction characteristic.

**Table 2:** **Fixed effects of dependency and perfectionism on recent stress and 4 different partner interaction characteristics**.

	**Dependency**		**Perfectionism**	
	***b***	***SE***	***b***	***SE***
**Recent stress**	0.084	0.06	0.006	0.035
**Partner interactions**				
Topic distress	0.051	0.026	−0.02	0.018
Conflict	0.003	0.025	0.025	0.016
Criticism	−0.018	0.018	0.024	0.014
Emotional support	0.016	0.019	−0.036^*^	0.015

*Statistically significant coefficients are shown in bold type*.

* p < 0.05.

### Primary analyses

#### Full-scale DAS-A

First we regressed each of our 3 dependent variables (depressed mood, overall well-being, and perceived support) on received emotional support, the full Dysfunctional Attitude Scale, and their interaction effect. We also entered our recent stress index as a covariate. The first horizontal section of Table 3 presents the fixed-effects for these models. Because our analyses require data from both the day of an interaction and the day after, analyses do not include all 235 reported interaction-days (due to missing data for the following day). Thus, all analyses involving next-day outcomes are *n* = 217, representing interactions from 56 individuals.

**Table 3:** **Regression coefficients for the effects of emotional support, dysfunctional attitudes, their interaction, and recent stress on change in mood and appraisals the day after a significant interaction with a partner**.

	**Outcomes on day *t* + 1**					
	**Depressed mood**		**Well-being**		**Perceived support**	
	***b***	***SE***	***b***	***SE***	***b***	***SE***
Previous day outcome^a^	0.283^***^	0.057	0.434^***^	0.065	0.26^***^	0.064
Recent-stress load	0.047^***^	0.013	−0.025	0.015	−0.044^**^	0.016
Emotional support	0.063	0.04	−0.038	0.048	−0.061	0.048
DAS-A full scale^b^	0.004^*^	0.002	−0.006^*^	0.002	−**0.005**^†^	0.003
DAS-A X Support	0.002	0.002	−0.001	0.002	−0.001	0.002
Previous day outcome	0.300^***^	0.056	0.483^***^	0.064	0.288^***^	0.065
Recent-stress load	0.049^***^	0.013	−**0.026**^†^	0.015	−0.045^**^	0.016
Emotional Support	0.036	0.041	−0.009	0.05	−0.03	0.05
DAS-A dependency	0.004	0.008	−0.008	0.008	−0.003	0.011
Dependency X Support	0.019^**^	0.007	−0.021^*^	0.009	−0.021^*^	0.009
Previous day outcome	0.238^***^	0.057	0.445^***^	0.065	0.258^***^	0.064
Recent-stress load	0.05^***^	0.013	−0.030^*^	0.015	−0.046^**^	0.016
Emotional support	0.074^†^	0.039	−0.047	0.047	−0.067	0.048
DAS-A perfectionism	0.012^*^	0.005	−0.015^*^	0.006	−0.016^*^	0.008
Perfectionism X Support	0.002	0.004	0.002	0.005	0.002	0.005

Statistically significant coefficients are shown in bold type.

a For each model, this variable is the value of the dependent variable (e.g., depressed mood) on day t.

b Due to the group-mean centering of emotional support, main effects for the dysfunctional attitudes scales are interpreted as the effect of dysfunctional attitudes on the outcome when a person receives his own average level of emotional support.

† *p < 0.10*,

* *p < 0.05*,

** *p < 0.01*,

*** p < 0.001.

Contrary to our hypotheses, the interaction effect between dysfunctional attitudes and emotional support was not significant for any of our outcomes. However, consistent with our secondary hypotheses, dysfunctional attitudes predicted increased depressed mood [*F*_(1, 55.38)_ = 4.22, *p* = 0.045] and reduced well-being [*F*_(1, 58.08)_ = 6.52, *p* = 0.013], and displayed a trend-level relationship with reduced perceived support [*F*_(1, 57.45)_ = 3.68, *p* = 0.06]. Emotional support did not predict any of the outcomes. As expected, in all 3 models the main effects of the previous day's outcome variables were significant, such that, for example, previous day depressed mood predicted next day-depressed mood. Recent stress also predicted greater depressed mood [*F*_(1, 195.12)_ = 12.06, *p* < 0.001] and reduced perceived support [*F*_(1, 204.92)_ = 7.07, *p* = 0.008], but not well-being [*F*_(1, 143.07)_ = 2.69, *p* = 0.10].

#### Dependency

Next we investigated whether the specific content and themes of a person's dysfunctional attitudes are important for understanding the relationship between dysfunctional thought, emotional support, and mood and appraisals. To do this we re-ran our models using the perfectionism and dependency subscales of the DAS-A. The fixed effects for dependency and perfectionism analyses are presented in the second two horizontal sections of Table 3.

**Figure 1:**
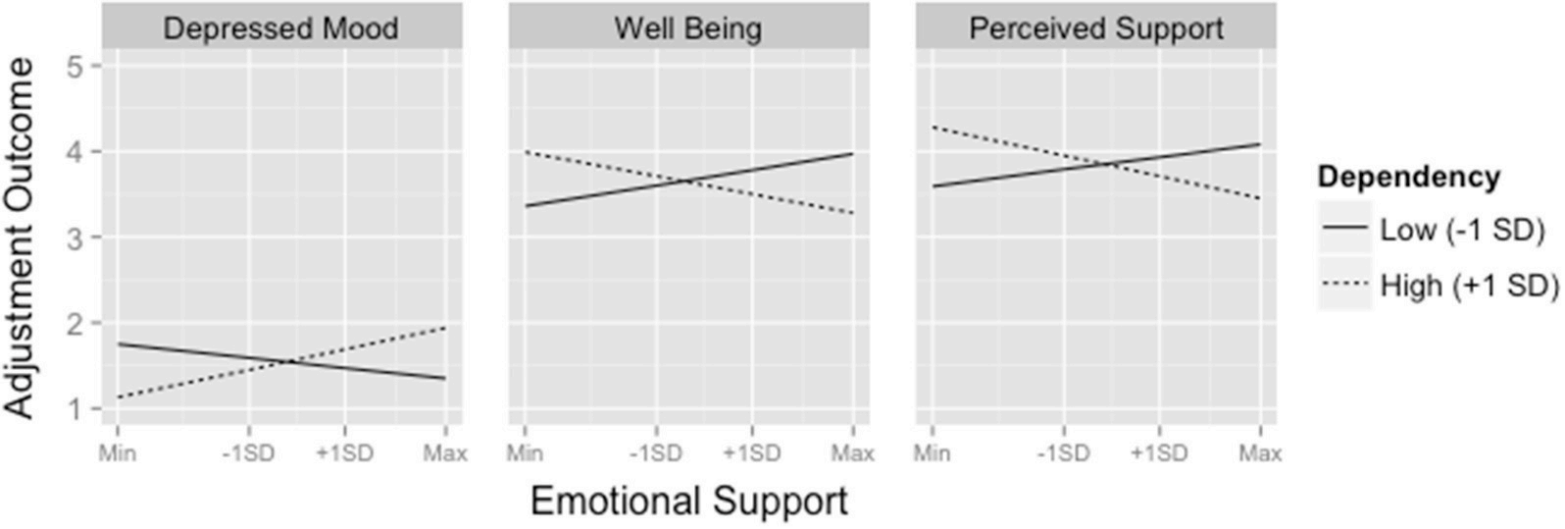
**Predicted values of depressed mood, well-being, and perceived support as a function of emotional support at 1 ***SD*** above and below the mean of dependency**. Adjustment outcomes were measured on a 1–5 Likert scale. To aid in interpretation of the group-mean centered Emotional Support variable, tick-marks on the x-axis are provided for ± 1 standard deviation away from individuals' means as well as the minimum and maximum values in the dataset.

As expected, previous day outcomes and recent stress both significantly predicted each dependent variable. Consistent with our moderation hypotheses, dependency interacted with emotional support to predict all 3 dependent variables. Simple slopes analyses revealed that, for people with more dependent attitudes, received emotional support predicted greater next-day depressed mood [*b* = 0.15, 95% CI (0.057, 0.27)], reduced well-being [*b* = −0.13, 95% CI (−0.30, −0.041)], and reduced perceived support [*b* = −0.15, 95% CI (−0.31, −0.034)]. For those with fewer dependent attitudes, emotional support did not significantly predict any of the outcomes at this level of dependency, as all of the 95% confidence intervals for the regression coefficients included zero: depressed mood [*b* = −0.07, 95% CI (−0.17, 0.035)], well-being [*b* = 0.11, 95% CI (−0.031, 0.26)], and perceived support [*b* = 0.09, 95% CI (−0.084, 0.24)]. Figure 1 illustrates the predicted values of each of these outcomes at high and low levels of dependency.

#### Perfectionism

Similar to findings from the full scale DAS-A, neither the main effect of emotional support nor the interaction effect between emotional support and perfectionism were significant in any of the models. Furthermore, perfectionism predicted all three of the dependent variables in expected directions: depressed mood [*F*_(1, 142.33)_ = 4.21, *p* = 0.046], well-being [*F*_(1, 47.42)_ = 25.56, *p* = 0.023], and perceived support [*F*_(1, 49.74)_ = 4.08, *p* = 0.049]. As expected, the prior day's outcome and recent stress also predicted all three dependent variables.

### Analyses of potential confounds

Dysfunctional attitudes are associated with greater exposure to and perceptions of stress. Thus, it is possible that greater stress exposure for people with higher levels of dependency may create spurious negative effects of emotional support. Specifically, higher stress exposure may lead simultaneously to greater received emotional support and reduced well-being. Such a scenario could create a spurious negative relationship between emotional support and subsequent well-being. In order to address part of this confound, we controlled for recent stress in each of our primary analyses. Still, it is important to clarify the relationship between stress exposure and emotional support across levels of dependency. To do this, we regressed emotional support on the interaction between recent stress and dependency. If the negative effects of receiving high amounts of emotional support are in fact spurious effects (due to higher amounts of stress leading to greater emotional support), then the interaction effect should be significant such that individuals with high levels of dependency should have a stronger, *positive* relationship between recent stress and emotional support. This was not the case: Not only is the interaction effect not significant [*F*_(1, 205.37)_ = 3.10, *p* = 0.08], higher levels of recent stress is related to *less* emotional support [*b* = −0.11, *F*_(1, 204.47)_ = 14.62, *p* < 0.001]. Thus, regardless of levels of dependency, people tend to report *less* emotional support when they are experiencing high levels of stress, making it unlikely that the negative effects of emotional support are due to greater simultaneous stress exposure and emotional support.

Because these data include a variety of types of interactions with varying levels of distress, we also wanted to evaluate whether the paradoxical effects of emotional support remained even when examining only those interactions concerning topics that were moderately to highly distressing. To do this, we re-ran our dependency analyses on a subset of couples' interactions that had distress ratings of 3, 4, or 5 (*n* = 145), corresponding to the anchors “moderately”, “very much”, and “extremely”. In these models, the interaction effect between dependency and emotional support was significant for depressed mood [*F*_(1, 114.35)_ = 5.01, *p* = 0.026] and perceived support [*F*_(1, 113.6)_ = 5.54, *p* = 0.02], and demonstrated trend level significance for well-being [*F*_(1, 125.37)_ = 3.77, *p* = 0.054]. Analyses of simple slopes using bootstrapped 95% confidence intervals indicated that at high levels of dependency, emotional support was significantly related to greater depressed mood and reduced well-being and perceived support. Emotional support was not related to any outcomes at lower levels of dependency.

## Discussion

In this study we evaluated the hypothesis that dysfunctional attitudes make individuals more vulnerable to the cognitive and emotional costs of received support. Specifically, we tested whether trait dysfunctional attitudes moderated the relationship between daily emotional support receipt and subsequent depressed mood, well-being, and perceived support. Contrary to this hypothesis, we did not find evidence that dysfunctional attitudes, as assessed by the full-scale DAS-A, altered the relationship between emotional support and subsequent well-being. However, we did find that both dysfunctional attitudes and recent stress exposure over the preceding 3 days were related to greater depressed mood and reduced well-being and perceived support. This is consistent with research and theory on dysfunctional attitudes that implicates them as a cognitive vulnerability for depression (Weissman and Beck, 1978; e.g., Kuiper et al., 1987).

We also sought to understand whether the specific themes of a person's dysfunctional attitudes (i.e., perfectionism vs. dependency) differentially affect the relationship between emotional support and subsequent mood and appraisals. We found that perfectionism did not moderate the relationship between emotional support and these outcomes but did predict more negative mood and appraisals, even after controlling for recent stress exposure. In contrast, dependency moderated the relationship between emotional support and all three outcomes such that individuals with greater dependency reported greater depressed mood and reduced well-being and perceived support as a function of increased emotional support. For individuals low in dependency, however, emotional support was not related to any subsequent outcomes.

These divergent findings related to perfectionism and dependency suggest that the consequences of receiving emotional support depend on the specific themes of a person's dysfunctional attitudes. Perfectionistic attitudes do not interact with emotional support. This may be because these attitudes, which entail high, self-imposed standards for oneself around performance and achievement, may not be affected by emotional support from a partner. Emotional support often entails attempts to raise a person's self-esteem, but these opinions of other people may do little to increase or decrease the salience of the one's own underlying attitudes.

Dependent attitudes, in contrast, do interact with emotional support. This may be because dependent attitudes reflect a heightened value of others' feelings and evaluations (e.g., “My happiness depends more on other people than it does on me,” “My value as a person depends greatly on what others think of me.”), as well as fear of criticism, rejection, and lack of support (e.g., “It is awful to be put down by people important to you,” “If others dislike you, you cannot be happy,” “If you don't have other people to lean on, you are going to be sad.”). Although those with dependent attitudes deeply value their partners' acceptance and support, these data suggest that receiving emotional support may paradoxically activate or exacerbate these concerns about what their partners think of them. This could be imagined in a number of scenarios: For instance, when a dependent individual discloses a personal failure, she may be prone to worrying how her partner's opinion of her has changed in light of this weakness and imperfection. This is consistent with research indicating that the presence of a supportive friend or relative during a stressful task can lead to heightened physiological arousal, likely due to the potential negative evaluations of the friend or relative (Allen et al., 1991). Support receipt may also create feelings of indebtedness (Gleason et al., 2008), which could subsequently arouse concerns about whether one's partner feels dissatisfied with an imbalanced relationship.

It is interesting to note that the interaction between dependency and emotional support predicts a seemingly global shift toward negative mood and appraisals, which might be surprising considering the specifically interpersonal nature of dependent attitudes. But these findings are consistent with research and theory on dysfunctional attitudes. Dysfunctional attitudes are considered a cognitive vulnerability to depression because in the presence of specific stressor, dysfunctional attitudes lead to broad patterns of negative, self-referent thinking (Beck, 1967; Weissman and Beck, 1978). For instance, Joiner et al. (1999) found that when individuals high in dysfunctional attitudes received a bad grade on a midterm exam, they demonstrated increases in depressive thinking across a variety of domains unrelated to performance, such as negative thoughts about one's attractiveness, one's relationships with others, and one's social value. In this study, the receipt of support by individuals with high dependency may have lead to more general depressive thinking, which was apparent in their more negative appraisals of well-being (which includes feelings of esteem, competence, control, and life satisfaction), appraisals of perceived support, and mood.

These findings have important implications for our understandings of emotional support and its relationship to depression vulnerability. First, the present study further supports the growing literature indicating that emotional support can have emotional costs (e.g., Bolger et al., 2000; Bolger and Amarel, 2007), usually rooted in negative appraisals about the self and relationships with others. These findings extend this literature both substantively and methodologically. Substantively, the present study identifies dependent attitudes as a specific factor that may modulate individuals' likelihood of experiencing negative moods and appraisals after emotional support receipt. Methodologically, we improve on previous diary studies by assessing the *extent* supportiveness and other important characteristics of *specific* couples' interactions. This approach captures more precise variation in the quality and nature of couples' interactions and allows us to rule out an important confound in prior studies: That negative aspects of couples' interactions, such as criticism that can sometimes accompany support, are the true source of negative outcomes. Lastly, by assessing the consequences of support in a variety of couples' interactions, our results may be generalizable to a wider array of couples' interactions. Thus, dependency and emotional supportiveness may influence a variety of couples' interactions, whether or not they qualify as support transactions or conflicts.

These findings suggest an, as yet, rarely hypothesized relationship between emotional support and cognitive vulnerability to depression: That receiving emotional support might often be bad for those with high levels of dependent dysfunctional attitudes. Although this finding is supported by some studies that have found positive relationships between received support and depression symptoms, on the whole, the literature relating received support to depression is decidedly mixed (Nurullah, 2012). These mixed findings may, at least in part, be due to differences in study design and measurement of received support. Most studies of depression and received support employ monthly checklists of received supportive behaviors. This approach is prone to numerous confounding issues, including recall bias and the fact that individuals who have the most problems are likely to be the recipients of the most support (Ibarra-Rovillard and Kuiper, 2011). Most importantly, this approach is unable to capture the immediate effects of support on mood and cognition. In contrast, those studies that measure or evaluate *individual* support transactions, for instance via laboratory observation (e.g., Howland and Simpson, 2010), laboratory experimentation, (e.g., Bolger and Amarel, 2007), or daily diary measurement (Bolger et al., 2000; Shrout et al., 2006; Gleason et al., 2008) have been more likely to detect negative effects of support, possibly because they can evaluate the immediate consequences of received support. Thus, although somewhat surprising, the findings presented in this study are in line with other methodologically similar studies, and they suggest a need for depression researchers to adopt designs that can capture costs of received support and evaluate their long-term consequences.

The present study has a number of limitations. The primary limitation of is its use of correlational data, which cannot demonstrate a causal relationship between emotional support and psychological well-being or between dysfunctional attitudes and emotional support. Consequently, we cannot definitively rule out the possibility of third variables that influence both received emotional support and well-being (e.g., stressful events preceding interactions) or of qualitative differences in the interactions of people with high and low dependency (e.g., greater criticism). Our statistical controls and supporting analyses, however, were not consistent with these alternatives. In our primary analyses, the moderation of emotional support by dependent attitudes held even while controlling for recent stressors and when confining our analyses only to interactions rated as moderately to extremely distressing. Furthermore, a person's level of dependency was unrelated to perceptions of criticism, conflict, or emotional support in the interaction and also unrelated to the level of distress toward the topic of couples' interactions.

The present study also cannot rule out how a partner's behavioral responses after providing support may affect participant mood. It is possible that dependent individuals are less accepting of support from their partners, and that this perceived rejection of one's support attempt may lead the support provider subsequently to withdraw and engage less positively with the recipient (Marigold et al., 2014). We were able to address this issue to some degree by controlling for stressors experienced on the day following couples' interactions. However, it is important that future studies replicate these findings in controlled experimental designs, which can eliminate these confounders.

Despite its limitations, the present study represents an important development in our understanding of cognitive vulnerability to depression and received support, as few studies have investigated how the costs of support affect vulnerability to depression. The present study supports the hypothesis that individuals with certain dysfunctional attitudes may be at greater risk of experiencing negative moods and thoughts after receiving high levels of emotional support from a partner. This vulnerability may result from a tendency to make negative appraisals about a partner's attitudes toward oneself (e.g., disapproval, rejection). Future studies can extend this finding by directly evaluating how support alters the salience of dysfunctional attitudes and the contexts in which this is most likely to occur. Furthermore, because the present study suggests that individuals already vulnerable to depression, by virtue of having dysfunctional attitudes, may be more likely to experience iatrogenic effects of support, it is important for future studies to examine whether frequent encounters with these costs accrue and increase risk for onset or relapse of depression.

## Author contributions

SF developed the study concept. Both authors contributed to the study design. Testing and data collection were performed by SF and research assistants, Matthew Yung, Matthew P. Abrams, and Meghan Brady. SF performed the data analysis and interpretation under the supervision of CH. SF drafted the paper and CH provided critical revisions. Both authors approved of the final version of the paper for submission.

### Conflict of interest statement

The authors declare that the research was conducted in the absence of any commercial or financial relationships that could be construed as a potential conflict of interest. The handling Editor declared a shared affiliation, though no other collaboration, with one of the authors SF and states that the process nevertheless met the standards of a fair and objective review.

## Footnotes

^1^In the analyses of recent stress, we analyzed only the recent stress index for the days after interactions (i.e., the same days as the outcome variables in our primary analyses). This allows a better assessment of whether highly dependent or perfectionistic people experience greater overall stress in the days before and after a significant partner interaction.
